# Oxytocin in the paraventricular nucleus attenuates incision-induced mechanical allodynia

**DOI:** 10.3892/etm.2015.2285

**Published:** 2015-02-11

**Authors:** YANFENG ZHANG, YONG YANG, RUPING DAI, HUI WU, CHANGQI LI, QULIAN GUO

**Affiliations:** 1 Department of Anesthesiology, Xiangya Hospital, Central South University, Changsha, Hunan 410078, P.R. China; 2 Department of Anesthesiology, The Second Xiangya Hospital, Central South University, Changsha, Hunan 410011, P.R. China; 3 Department of Medical Oncology, Hunan Provincial Tumor Hospital, The Affiliated Tumor Hospital, Xiangya School of Medicine, Central South University, Changsha, Hunan 410013, P.R. China; 4 Department of Anatomy and Neurobiology, Xiangya School of Medicine, Central South University, Changsha, Hunan 410078, P.R. China

**Keywords:** oxytocin, incisional pain, mechanical allodynia, supraspinal analgesia, paraventricular nucleus, supraoptic nucleus, spinal cord

## Abstract

Oxytocin (OT) neurons localized in the paraventricular nucleus (PVN) and supraoptic nucleus (SON) send fibers to the brain and spinal cord. While most previous studies have looked at the role of OT in chronic pain, few have investigated the role of OT in acute pain, particularly postoperative pain. In the present study, the role of OT in incision-induced allodynia was explored for the first time, using a rat incisional pain model. Immunohistochemical staining showed that, compared with the baseline (prior to incision) measurements, the OT content in the PVN was significantly decreased at 0.5, 1.0 and 3.0 h post-incision and returned to the baseline level at 6.0 h post-incision. By contrast, there was no significant difference in the OT content in the SON prior to and subsequent to incision. A dose-dependent inhibition of mechanical hypersensitivity was detected 30 min after intracerebroventricular injection of OT (100, 400 or 600 ng) and lasted for 3.0 h. No significant difference was noted, however, between the intrathecal OT injection group (600 ng) and the control group. In conclusion, the present study provides the first *in vivo* evidence that OT in the PVN predominantly attenuates incision-induced mechanical allodynia at the supraspinal, rather than the spinal, level. This suggests that OT is involved in supraspinal analgesia for postoperative pain.

## Introduction

The neuropeptide oxytocin (OT) is synthesized within the hypothalamus in the paraventricular nucleus (PVN) and supraoptic nucleus (SON) (1). Central OT plays an important role in modulating a variety of physiological functions in mammals, including parturition, lactation, social behavior and memory (2).

Accumulating evidence shows that OT has analgesic properties as a neurotransmitter or neuromodulator (3). Anatomically, OT neurons in the PVN send direct descending fibers to several brain regions involved in pain perception (4), including the dorsal and ventral hippocampus, amygdala, periaqueductal gray (PAG) and raphe nuclei, as well as the superficial dorsal horn of the spinal cord (1,5). In addition, extensive clinical and preclinical studies have demonstrated that OT in the PVN, rather than that in the SON, is involved in anti-nociception or analgesia (6,7). It has been reported that the intrathecal injection of OT can significantly reduce withdrawal responses to mechanical and cold stimulation in sciatic nerve-ligated rats, and the effect can be blocked by intrathecal injection of an OT antagonist (6,7). In humans, Madrazo *et al* (8) reported that the intractable thoracic pain of a patient with diffuse mesothelioma could be reduced by 88% for 77 min by intracerebroventricular injection of OT. Yang (9) reported that acute and chronic lower back pain in humans resulted in a marked change of OT content in the cerebral spinal fluid and plasma; OT could relieve this lower back pain, and the effect of OT was blocked by an OT antagonist.

While most previous studies have looked at the role of OT in chronic pain, few investigations have been carried out into the role of oxytocin in acute pain, particularly postoperative pain. In the present study, the role of OT in incisional-induced allodynia was explored for the first time, to the best of our knowledge.

## Materials and methods

### Animals

The experiments were performed using male Sprague Dawley rats (200–250 g) provided by the Animal Experimental Center of Xiangya School of Medicine, Central South University (Changsha, China). The rats were housed singly with food and water available *ad libitum* in a temperature-controlled (25±2°C) room and under a 12-h light/dark cycle. All experiments were conducted in accordance with the guidelines of the International Association for the Study of Pain (10). Every effort was made to minimize any suffering of the animals and the number of animals used.

### Incisional pain model

The hindpaw incision model was performed as described by Brennan *et al* (11). The model and control groups each consisted of 30 mice. Briefly, rats were anesthetized with 2% isoflurane (Sigma-Aldrich, St. Louis, MO, USA) delivered through a nose cone. The plantar aspect of the right hindpaw was prepared in a sterile manner with a 10% povidone-iodine solution (Sigma-Aldrich), and the paw was positioned through a hole in a sterile drape. A 1-cm longitudinal incision was made through the skin and fascia of the plantar aspect of the foot, with a size 11 blade, starting at a point 0.5 cm away from the proximal edge of the heel and extending toward the toes. The plantaris muscle was raised and incised longitudinally, with its origin and insertion remaining intact. The incision was closed with two mattress sutures of 5-0 nylon. Following surgery, the animals were allowed to recover in their home cages. Sham control groups, consisting of rats that received anesthesia, antiseptic preparation and topical antibiotic without an incision, were used in the study.

### Intracerebroventricular injection of OT

Animals were anesthetized by intraperitoneal injection of chloral hydrate (300 mg/kg) and mounted on a stereotaxic frame. A stainless steel guide cannula (0.8 mm outer diameter) was directed into the lateral ventricle (anteroposterior, −0.92 mm; lateral, 1.3 mm; dorsoventral, 4.0 mm) according to the rat brain stereotaxic atlas of Paxinos and Watson (12). The cannula protruded 1 cm above the skull and was fixed to the skull by dental acrylic. Finally, the opening was sealed. If no abnormality was noted during the first three days after implantation, a stainless steel needle (0.4 mm diameter) was inserted directly into the lateral ventricle along the guide cannula and 10 *μ*l vehicle (0.9% saline) or OT (100, 400 or 600 ng; Sigma-Aldrich) solution was injected over 10 min immediately after hindpaw incision.

### Intrathecal injection of OT

Intrathecal catheter implantation was performed as previously described (13), with minor modifications. Following incision, OT (600 ng, total volume 10 *μ*l) was injected intrathecally through the catheter, followed by a 10-*μ*l saline flush, and mechanical hypersensitivity was assessed.

### Mechanical hypersensitivity assay

Von Frey filaments (Stoelting Co., Wood Dale, IL, USA) were used to assess the withdrawal threshold prior to incision and at 0.5, 1.0, 3.0, 6.0, 24.0 and 72.0 h after incision, according to the ‘up-down’ algorithm, as previously described (14). The assay was performed by an investigator blinded to the treatment. At the end of the experiments, 20 *μ*l methylthionine chloride was injected into the lateral ventricle via the cannula, and a coronal section was made across the lateral ventricle. Only data from rats whose ventricle system was filled with methylthionine chloride were included in the analysis.

### Immunohistochemistry

Rats were deeply anesthetized with chloral hydrate (300 mg/kg) and perfused transcardially with 4% paraformaldehyde. The brain and lumbar enlargement from each rat were post-fixed with 4% paraformaldehyde for 4 h, and then immersed overnight in 0.01 M phosphate buffer containing 30% sucrose. Cryostat-cut brain and lumbar spinal cord sections (30-*μ*m) were incubated overnight at room temperature with polyclonal rabbit anti-human anti-OT antibody (#O4389; 1:2,000 dilution; Sigma-Aldrich). Subsequent to washing in 0.01 M phosphate-buffered saline, the sections were incubated with biotinylated polyclonal goat anti-rabbit secondary antibody (#A6154; 1:5,000 dilution; Sigma-Aldrich) followed by an avidin-biotin-peroxidase complex (Sigma-Aldrich) for a further 2 h, prior to being visualized with diaminobenzidine. Finally, the sections were mounted on slides (in the dark for immunofluorescence) and analyzed under a microscope (IX83; Olympus Corporation, Beijing, China). Negative controls were set up by performing the experiments without the primary antibodies.

### Evaluation of immunostaining

Image-Pro Plus 6.0 software (Media Cybernetics Inc., Silver Spring, MD, USA) was used to quantify the optical density value of OT in the PVN and SON and lamina I/II of the superficial dorsal horn of the lumbar enlargement. According to the rat brain stereotaxic atlas of Paxinos and Watson (12), the PVN and SON were located from 1.08 to 2.16 mm and from 0.92 to 1.44 mm posterior to the Bregma, respectively (15). The mean optical density of the OT staining was calculated by dividing the optical density with the measurement area.

### Statistical analysis

Statistical analyses were performed using SPSS software, version 11.0 (SPSS Inc., Chicago, IL, USA). Comparisons of means between two groups were performed with Student’s t-tests and among multiple groups with one-way analysis of variance followed by post hoc pairwise comparisons using Dunnett’s tests. Data are presented as the mean ± standard error of the mean. A two-tailed P<0.05 was considered to indicate a statistically significant difference in this study.

## Results

### Hindpaw incision induces a reduction in OT levels in the PVN

**Figure 1:**
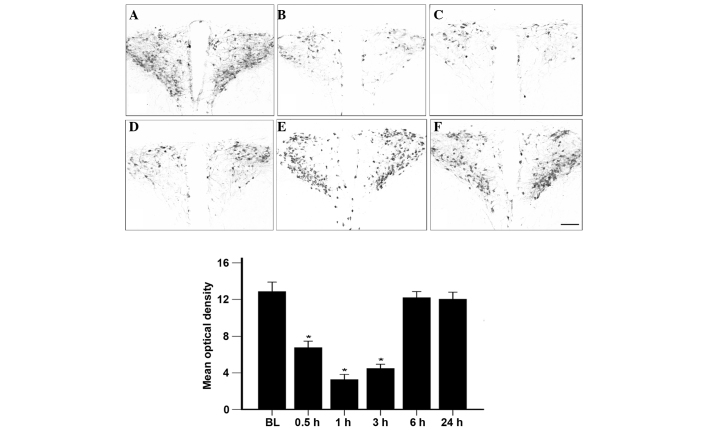
OT content in the PVN prior and subsequent to hindpaw incision. Immunohistochemical staining for OT was performed in the PVN in rats at (A) BL (prior to incision); and (B) 0.5 h; (C) 1.0 h; (D) 3.0 h; (E) 6.0 h and (F) 24.0 h after incision. The OT content was quantified using the optical density of the OT staining, which is shown in the bar graph. Data are presented as the mean ± standard error of the mean. Scale bar, 200 μm. Magnification, ×200. ^*^P<0.05 vs. BL. OT, oxytocin; PVN, paraventricular nucleus; BL, baseline.

To determine whether surgical incision affected OT in the PVN, OT in the PVN of animals with or without hindpaw incision was immunostained and the OT content was quantified using the optical density of the OT staining. As shown in Fig. 1, the OT content (optical density value) in the PVN at baseline (prior to incision) was 13.5±1.2 (n=7). Following incision, the OT content in the PVN was 6.7±1.0, 3.5±0.8 and 4.8±0.9 at 0.5, 1.0 and 3.0 h, respectively (n=6 at each time-point), indicating a significant decrease compared with the baseline (P<0.05). Between 6 and 24 h after incision, the OT content in the PVN returned to the baseline level (n=6 at each time-point) (Fig. 1). By contrast, the sham groups (n=5 at each time-point) showed no significant differences in the OT content in the PVN prior to and subsequent to incision (data not shown).

### Expression of OT in the SON and spinal cord remains unchanged following hindpaw incision

**Figure 2:**
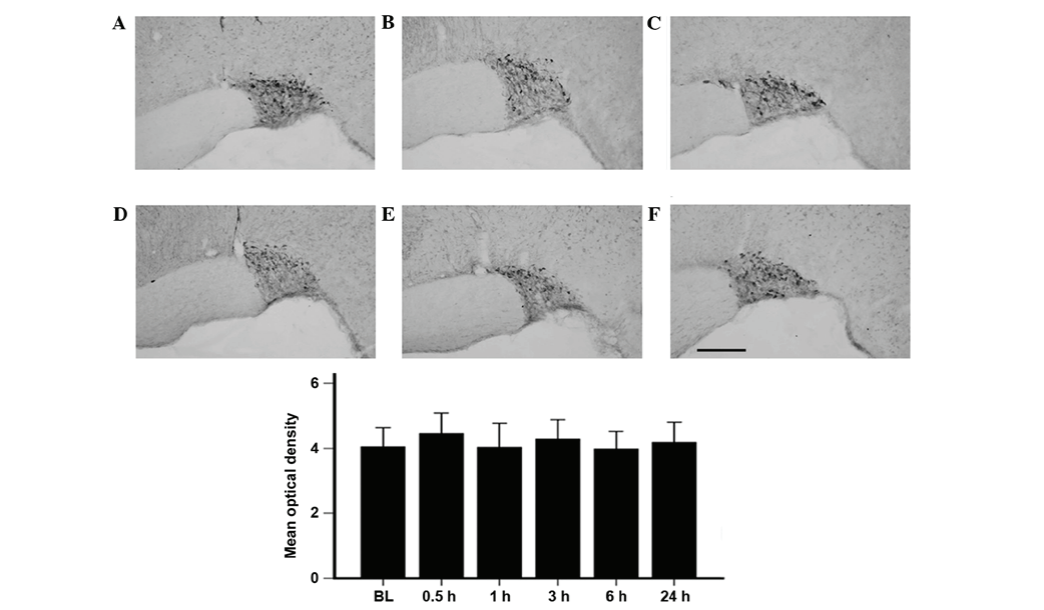
OT content in the SON prior and subsequent to hindpaw incision. Immunohistochemical staining for OT was performed in the SON in rats at (A) BL (prior to incision); and (B) 0.5 h; (C) 1.0 h; (D) 3.0 h; (E) 6.0 h and (F) 24.0 h after incision. The OT content was quantified using the optical density of the OT staining, which is shown in the bar graph. Data are presented as the mean ± standard error of the mean. Scale bar, 200 μm. Magnification, ×200. BL, baseline, OT, oxytocin; SON, supraoptic nucleus.

**Figure 3:**
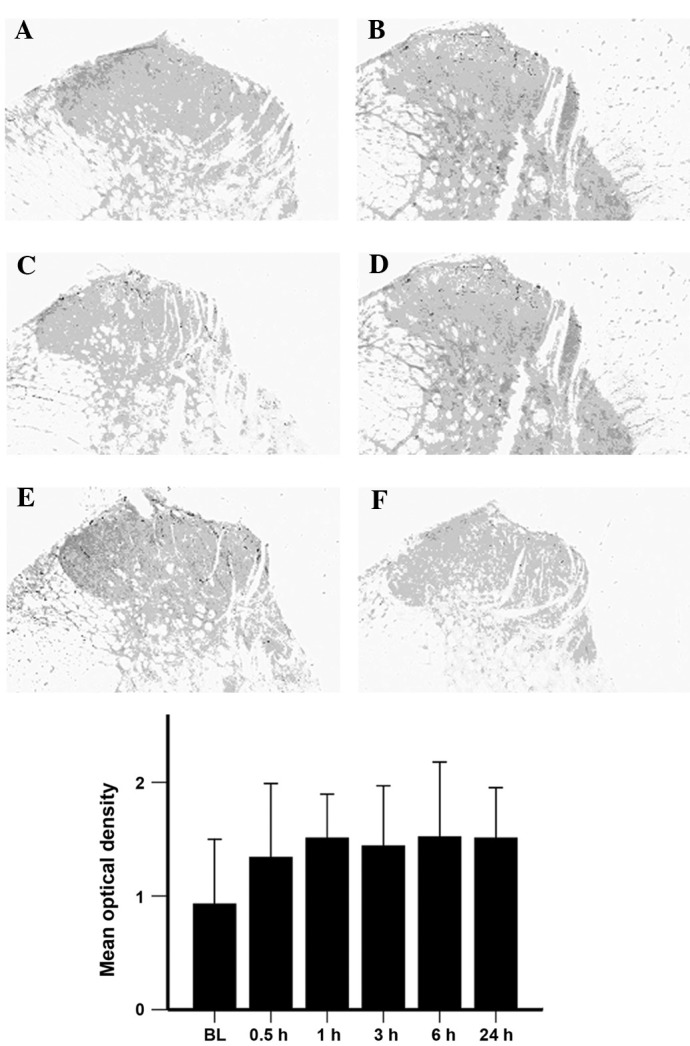
OT content in the spinal dorsal horn prior and subsequent to hindpaw incision. Immunohistochemical staining for OT was performed in the spinal dorsal horn in rats at (A) BL (prior to incision); and (B) 0.5 h; (C) 1.0 h; (D) 3.0 h; (E) 6.0 h and (F) 24.0 h after incision. The OT content was quantified using the optical density of the OT staining, which is shown in the bar graph. Data are presented as the mean ± standard error of the mean. Scale bar, 200 μm. Magnification, ×200. BL, baseline; OT, oxytocin.

As shown in Fig. 2, there was no significant difference in the OT content in the SON prior to and subsequent to incision. Notably, the dorsal horn of the spinal cord showed no significant difference in the OT content prior to and following incision, despite being an important center of pain modulation (Fig. 3).

### Intracerebroventricular but not intrathecal injection of OT attenuates mechanical hypersensitivity following hindpaw incision

**Figure 4:**
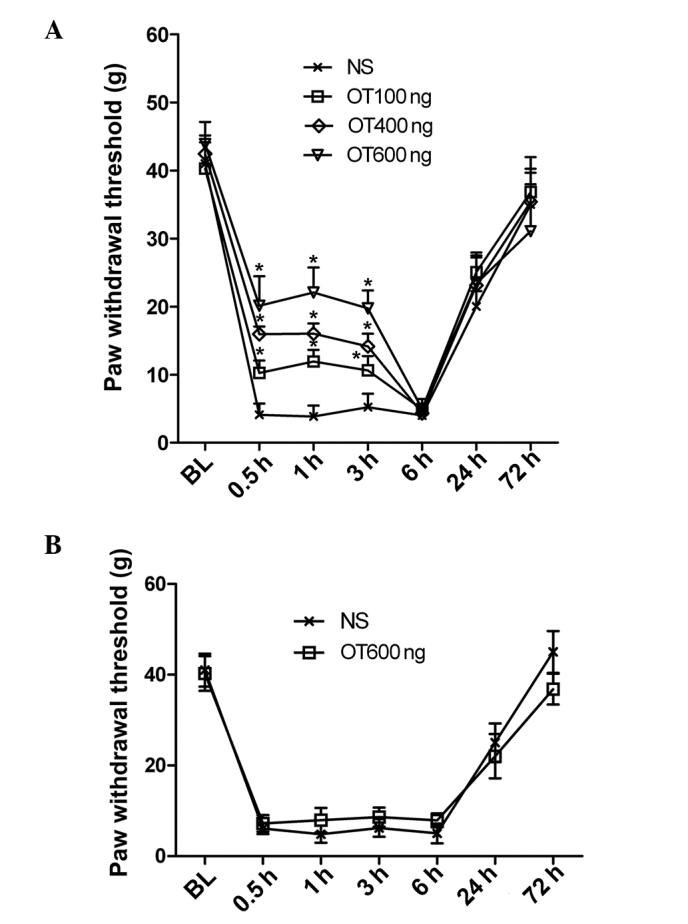
Effect of OT on incision-induced mechanical hypersensitivity of the hindpaw. The paw withdrawal threshold of the rats at BL (prior to incision) and 0.5, 1.0, 3.0, 6.0, 24.0 and 72.0 h after incision was measured with (A) intracerebroventricular injection of NS or 100, 400 or 600 ng OT or (B) intrathecal injection of NS or 600 ng OT. ^*^P<0.05 vs. NS. OT, oxytocin; BL, baseline; NS, normal saline.

To explore the role of OT in mechanical hypersensitivity induced by incision, intrathecal or intracerebroventricular injections of OT were performed immediately subsequent to hindpaw incision. A dose-dependent inhibition of mechanical hypersensitivity was detected 30 min after intracerebroventricular injection of OT (100, 400 or 600 ng) and lasted for 3.0 h (n=5 per group) (Fig. 4A). By contrast, no significant difference was noted between the intrathecal OT injection group (600 ng) and the control group (n=5 per group) (Fig. 4B).

## Discussion

In the present study, it was shown that OT is involved in the response to incisional pain at the supraspinal level, but not at the spinal cord as expected. Following incision, a marked decrease in the OT content was observed in the PVN; however, no significant change in the OT content was noted at the spinal cord within 24.0 h of the incision. Administration of exogenous OT at the supraspinal level by intracerebroventricular injection attenuated mechanical hypersensitivity in a dose-dependent manner, while injection at the spinal level did not produce any analgesia. These results are noteworthy, given that the spinal cord is an important center of pain modulation.

OT can exert a wide spectrum of central and peripheral effects, including modulation of the neuroendocrine reflex and analgesia (1,4). OT is synthesized in the PVN and SON and then transported to the peripheral circulation system or different regions of the central nervous system (1,4). In the present study, it was found that the OT content in the PVN was significantly reduced between 0.5 and 3.0 h after incision and returned to the baseline level after 6.0 h. The OT content in the SON, however, was not altered significantly following incision. This is in agreement with the observation by Rousselot *et al* (16) that the OT that is involved in an anti-nociceptive or analgesic response is likely to stem from the PVN and not the SON.

OT fibers project to several regions involved in pain modulation in the nervous system (17,18). It has been reported that ≥25% of OT-positive neurons in the PVN directly project to the superficial dorsal horn of the spinal cord (19). Furthermore, the spinal cord has overlap between OT fiber projections and the distribution of OT binding sites (6). Intrathecal injection of OT and electrical stimulation of the PVN can reduce the hypersensitivity of neuropathic or nociceptive responses in normal rats, and such effects can be inhibited by pretreatment with intrathecal injection of an OT antagonist (6). Condés-Lara *et al* (20,21) suggested that intrathecal injection of OT could reduce the activation of primary Aδ and C fibers evoked by somatic stimulation or neuropathy. Yu *et al* (22) found that intrathecal administration of OT dose-dependently attenuated carrageenan-induced inflammatory pain. In the spinal cord, OT transported from the PVN activates γ-aminobutyric acid (GABA)-ergic interneurons, which in turn inhibit the activation of glutamatergic primary sensory neurons. Such presynaptic inhibition prevents the transmission of nociceptive signals to the brain (23–25). In addition to the increase in GABA release, changes in the reversal potential of GABAergic currents in nociceptive neurons contribute to the analgesic effects of OT (26–29). In combination, the previous studies indicate that OT contributes to pain relief at the spinal level; however, in the present study, it was found that the expression of OT in the ipsilateral lumbar enlargement of the spinal cord remained unchanged until 24 h after incision, and intrathecal injection of OT did not improve mechanical hypersensitivity. By contrast, intracerebroventricular injection of OT dose-dependently elevated the mechanical hypersensitivity threshold. As intrathecal injection acts at the spinal cord, the results from the present study suggest that OT attenuates acute incisional pain at the supraspinal level rather than at the spinal level (30). The PVN sends projections to numerous brain regions implicated in pain modulation, such as the PAG, raphe magnus and dorsal raphe nuclei (31–33). Injection of OT into the cerebral ventricle can elevate pain thresholds in rats and humans (8,34). In addition, a previous study reported that OT could exert analgesic effects in neonatal rats during delivery, even when the descending OT fibers were cut by decerebration at the upper pons level (35). This suggests that OT in the brain can exert analgesic effects independently of the spinal cord and corroborates the findings from the present study.

The reduction of the OT content in the PVN following incision could be due to an alteration of OT transportation in the brain, as the OT content in the PVN was restored to the baseline level within 6 h of the incision. We aim to explore the underlying mechanisms in future studies.

In conclusion, the present study provides the first *in vivo* evidence, to the best of our knowledge, that OT in the PVN attenuates incision-induced mechanical allodynia at the supraspinal, but not the spinal, level. This suggests that OT is involved in supraspinal analgesia for postoperative pain.
